# Insights into the mechanisms of non-coding RNAs’ implication in the pathogenesis of Alzheimer’s disease

**DOI:** 10.17179/excli2022-5006

**Published:** 2022-06-30

**Authors:** Majid Khodayi-Shahrak, Mohammad Khalaj-Kondori, Mohammad Ali Hosseinpour Feizi, Mahnaz Talebi

**Affiliations:** 1 Department of Animal Biology, Faculty of Natural Sciences, University of Tabriz, Tabriz, Iran; 2 Neurosciences Research Center, Tabriz University of Medical Sciences, Tabriz, Iran

**Keywords:** Alzheimer's disease, miRNAs, lncRNAs, marker

## Abstract

Non-coding RNAs including long non-coding RNAs (lncRNAs) and microRNAs (miRNAs) are implicated in the regulation of gene expression at transcriptional, posttranscriptional, and epigenetic levels. Several studies in cell lines, animal models, and humans, have revealed that non-coding RNAs play crucial roles in the pathogenesis of Alzheimer's disease (AD). Detailed knowledge on their mechanism of implication in the AD pathogenesis can help to develop novel therapeutic and disease management strategies. The two main pathological hallmarks of AD are amyloid plaques resulting from the β-amyloid accumulation, and neurofibrillary tangles (NFT) due to the phosphorylated tau accumulation. Several lncRNAs and miRNAs play crucial roles in both these hallmarks of the AD pathogenesis and other AD-related pathological procedures such as neuronal and synaptic plasticity, neuroinflammation, neuronal differentiation and neuronal apoptosis. In this review, we outlined the non-coding RNAs and further discussed how they are implicated in these AD-related pathological procedures.

## Introduction

Alzheimer's disease (AD) is a devastating neurodegenerative disorder and the most common cause of dementia, accounting for approximately 60 % to 80 % of cases among the elderly worldwide (Doxtater et al., 2020[30]; Zhang and Wang, 2021[139]). AD is characterized by clinical features such as memory loss, cognitive impairment and behavioral function decline (Zhao et al., 2017[145]). The hallmark pathologies of Alzheimer's disease are the existence of extracellular plaques composed of amyloid-beta (Aβ) peptide resulting from abnormal proteolytic cleavage of amyloid precursor protein (APP) and intracellular neurofibrillary tangles (NFT), which result from hyper phosphorylation of the microtubule-associated tau proteins (Hoogmartens et al., 2021[54]; Mattson, 2004[92]). The cause and molecular mechanism of AD pathogenesis is very complex (Fotuhi et al., 2020[39]; Talebi et al., 2020[117]), and a couple of coding and non-coding genes and their polymorphisms are involved in AD development and progress (Mardokh Rohani et al., 2020[88]; Zhao et al., 2019[146]). Currently, the clinical diagnosis of AD is made by imaging and the measuring of Aβ levels in cerebrospinal fluid (CSF) during later clinical stages of AD (Cheng et al., 2015[22]; Mangialasche et al., 2010[86]). Thus, reliable biomarkers for early diagnosis are required to halt the disease development and improve the effects of therapeutic strategies. Genome wide analyses has revealed that only 2 % of the mammalian genome is made up of protein-coding genes (Wei et al., 2018[127]), and according to the ENCODE, most of what was already considered as nonfunctional or junk DNA is transcribed into non-coding RNAs (ncRNAs) (Ahmadi et al., 2020[1]; Ecker et al., 2012[32]). The ncRNAs can be classified either as housekeeping or regulatory subgroups. The former group plays crucial roles in functional homeostasis, whereas the latter are involved in regulation of genes. Regulatory ncRNAs can further be divided into ncRNAs with a length of fewer than 200 nucleotides (microRNAs, miRNAs, small interfering RNAs, and siRNAs), and the lncRNAs which contain more than 200 nucleotides and lacking the potential to be translated into proteins (Hombach and Kretz, 2016[53]; Idda et al., 2018[57]). Increasing evidence has suggested that ncRNAs are involved in the pathogenesis of AD (Wang et al., 2018[126]; Zhang et al., 2018[141]). In this review, we discuss both lncRNAs and microRNAs that are associated with Alzheimer's disease and provide new insights into our understanding of the etiology and pathophysiology of the disease.

## Characteristics of lncRNAs

**Figure 1:**
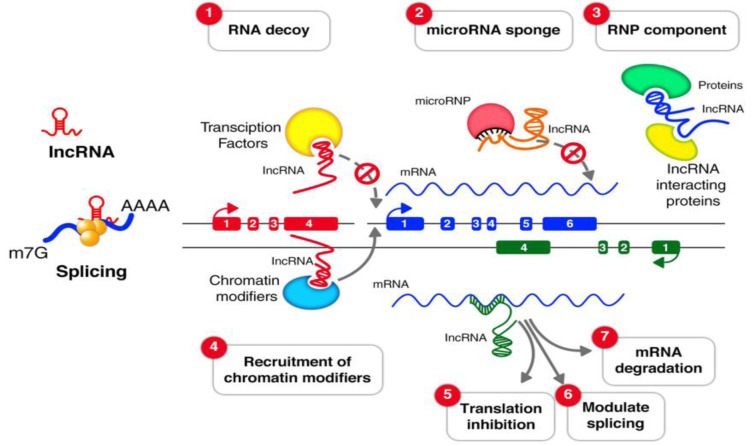
The lncRNA mechanisms of action. (1) RNA decoys; lncRNAs can act as decoys for transcription factors and sequester them away from their targets. (2) microRNA sponge; LncRNAs can bind to microRNAs as sponges to prevent them from their regulatory function. (3) Ribonucleoprotein (RNP) component; LncRNAs can play scaffolding roles to aid in the formation of Ribonucleoprotein (RNP) complexes. (4) Recruitment of chromatin modifiers; LncRNAs can recruit different kind of protein components which change the patterns of chromatin organization. LncRNAs can modulate (5) translation inhibition (6), splicing (7) and mRNA degradation.

Long non-coding RNAs (lncRNAs) comprise a class of endogenous regulatory RNA molecules that are over 200 nucleotides (nt) in length, without protein-coding capacity, and make up the largest proportions of the mammalian non-coding transcriptome (Ma et al., 2013[84]; Sun and Kraus, 2015[116]). Like protein-coding genes, lncRNAs are frequently transcribed by RNA polymerase II, contain 5ʹ end cap, may be polyadenylated or non-polyadenylated, and are located within nucleus or cytoplasm (Laurent et al., 2015[71]; Wei et al., 2018[127]). LncRNAs have a specific secondary structure that provides multiple sites for interaction with DNA, RNA and proteins (Wei et al., 2018[127]). In contrast to mRNAs, lncRNAs are expressed at lower levels, contain fewer exons and have attenuation sequence conservation across species (Cabili et al., 2011[20]; Yao et al., 2019[132]). The number of lncRNAs have been estimated from 59,000 to 102,000 listed in the NONCODE database (Iyer et al., 2015[59]; Jandura and Krause, 2017[60]). LncRNAs are not considered to be the dark matter, rather, they can control gene expression during a variety of cellular processes (Luo and Chen, 2016[83]), such as development (Roberts et al., 2014[107]), differentiation, genome rearrangement, and metabolism (Khorkova et al., 2015[65]; Zhu et al., 2013[151]). They also participate in chromatin modification regulation of the cell cycle and genetic imprinting (Goff and Rinn, 2015[43]; Kazemzadeh et al., 2015[62]). Besides, lncRNAs can impact gene expression at transcriptional, epigenetic, and post-transcriptional levels (Bhat et al., 2016[16]; Khajehdehi et al., 2021[63]) by involving in the splicing of RNA, messenger RNA (mRNA ) decay, and mRNA stability (Quinn and Chang, 2016[104]). A schematic view of the lncRNA function is outlined in the Figure 1(Fig. 1). 

## lncRNAs involved in Alzheimer’s Disease

**Figure 2:**
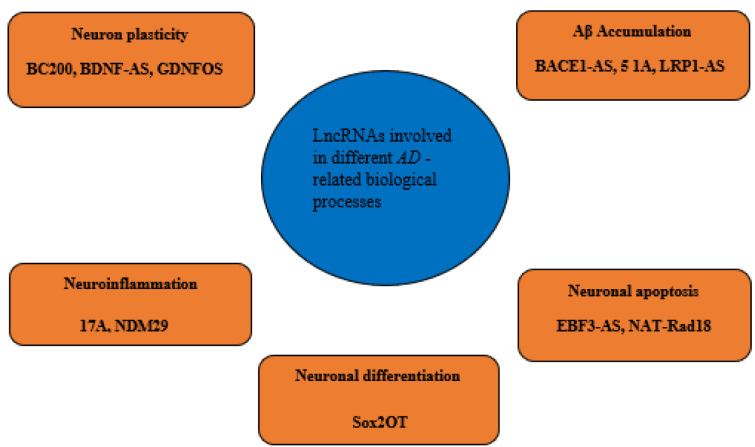
LncRNAs involve in different pathological aspects of Alzheimer's disease. Each AD-related lncRNA is grouped according to the involved AD-leading pathological procedure.

It was reported that up to 40 % of differentially expressed lncRNAs in the human genome are specific to the brain (Derrien et al., 2012[28]), and the aberrant lncRNA expression is implicated in the pathogeneses of many diseases, including cancer, cardiovascular and neurodegenerative diseases such as AD (Kwok and Tay, 2017[69]; Wan et al., 2017[121]). Transcriptome profiling analyses have identified that the lncRNA expression patterns appear disrupted in the brains of human and rat AD models compared to those in normal subjects (Crist et al., 2021[25]; Tang et al., 2019[118]). Annese and colleagues used RNA sequencing to assess changes in the lncRNA expression profiles of LOAD patients and observed that the expressions of 47 lncRNAs were significantly deregulated in the hippocampal region of LOAD patients (Annese et al., 2018[7]). Being differentially expressed in AD patients or animal models, lncRNAs can be considered as biomarkers and therapeutic targets for AD. At the molecular level, lncRNAs impact AD pathogenesis via a variety of regulatory mechanisms, including transcriptional, posttranscriptional and post-translational regulation (Luo and Chen, 2016[83]; Zhang, 2016[144]). Several lncRNA are known to implicate in synaptic impairment, APP processing, Aβ production and inflammation, the major causal factors in AD (Mufson et al., 2015[94]; Yang et al., 2017[130]). The most studied lncRNAs involving in AD are outlined in Table 1(Tab. 1) (References in Table 1: Ciarlo et al., 2013[24]; Faghihi et al., 2010[35]; Gao et al., 2020[40]; Gu et al., 2018[46]; Guo et al., 2018[47]; Huang et al., 2020[56]; Li et al., 2018[74], 2020[75][76]; Luo and Chen, 2016[83]; Magistri et al., 2015[85]; Massone et al., 2011[90]; Parenti et al., 2007[102]; Wang et al., 2018[123]; Yamanaka et al., 2015[129]; Yi et al., 2019[133]; Zhang et al., 2016[142], 2019[141]; Zhou and Xu, 2015[149]). LncRNAs contribute to the AD development by affecting different AD-related pathological procedures, including Aβ accumulation, neuron plasticity, neuroinflammation, neuronal differentiation and neuronal apoptosis. In this regard, we categorized the AD-related lncRNAs and outlined them in Figure 2(Fig. 2). In the following sections, we discuss these lncRNAs and how they are involved in each of the AD-related pathological procedures. 

## lncRNAs Modulate Aβ Accumulation in AD

### BACE1-AS 

Beta-secretase 1 (BACE1) is an enzyme central to the pathology of AD that contributed to the formation of Aβ peptides and extracellular plaque deposition (Chen et al., 2019[21]). BACE1 antisense transcript (BACE1-AS) is a conserved lncRNA that is mapped on the chromosome 11, transcribed in an antisense orientation regarding to the BACE1 locus, and can positively regulate both the BACE1 mRNA and its protein expression (Faghihi et al., 2008[34]). BACE1-AS is elevated in AD patients and in the APP transgenic mice (Faghihi et al., 2008[34]; Singer et al., 2005[111]). Binding of lncRNA BACE1-AS to the BACE1 mRNA increases the stability of BACE1 mRNA, and results in the high expression of BACE1 protein (Faghihi et al., 2008[34], 2010[35]; Wei et al., 2018[127]). Furthermore, cell stresses such as Aβ1-42 stimulation, high glucose, serum starvation, and H_2_O_2_ increase the expression of lncRNA BACE1-AS, which in turn makes BACE1 mRNA more stable, leading to enhanced APP processing and toxic Aβ production by a post-transcriptional feed-forward mechanism (Faghihi et al., 2008[34]). Production of more Aβ can in turn affect the up-regulation of BACE1-AS, leading to the formation of more plaques in the brain of AD patients, and finally aggravating the disease development (Faghihi et al., 2008[34]) (Figure 2(Fig. 2)). Liu et al. observed that silencing of the lncRNA BACE1- AS expression with short interfering RNA (siRNA) in SH-SY5Y cells attenuated the beta-secretase cleavage of APP and reduced the formation of the senile plaque in the cells (Liu et al., 2014[79]). Besides, knockdown of lncRNA BACE1-AS by siRNA alleviated the disease symptoms in AD animal models, including memory and learning behaviors (Singer et al., 2005[111]). BACE1-AS is upregulated in plasma of AD patients, suggesting that it may serve as a potential blood-based biomarker for AD development (Feng et al., 2018[37]; Fotuhi et al., 2019[38]).

### 51A 

The neuronal sortilin-related receptor 1(SORL1) gene encodes SORLA protein, which is involved in the processing of amyloid-β protein precursor (APP) and decrease Aβ peptides production in neurons (Andersen et al., 2005[6]). Studies have demonstrated that low levels of SORL1 in AD patients enhance the amyloidogenic process (Nicolas et al., 2018[99]; Scherzer et al., 2004[109]). SORL1, as a sorting receptor for APP and Aβ, interacts with cytosolic adaptor to guide APP between the trans-Golgi network and early endosome, where it affects trafficking and proteolytic processing (Reitz, 2012[106]). Decreased SORL1 expression might shift APP to the β-secretase cleavage pathway, increasing secreted APP production and subsequent Aβ production (Reitz, 2012[106]). The lncRNA 51A is located on the antisense orientation to the intron 1 of SORL1 gene (Yin et al., 2015[134]), and in contrast to the SORL1, the expression of lncRNA 51A is upregulated in the brain of AD cases. Mechanistically, lncRNA 51A changes the splicing pattern of SORL1 to an alternatively spliced short form which is associated with the abnormal APP processing, and promotes increased Aβ production (Ciarlo et al., 2013[24]). Furthermore, Deng et al. found that lncRNA 51A is increased in the plasma of AD patients, indicating its possible involvement in the pathobiology of AD (Chen et al., 2019[21]; Deng et al., 2017[26]).

### LRP1-AS

Low density lipoprotein receptor-related protein 1(LRP1), as a receptor is involved in a variety of cellular processes including cell signaling, lipoprotein metabolism, and APP trafficking and processing (von Einem et al., 2010[120]). LRP1 is abundantly expressed in different cell types in the brain including neurons, vascular and glial cells (Auderset et al., 2016[10]; Bruno et al., 2010[18]). LRP1 plays a pivotal role in the beta-amyloid uptake and beta-amyloid clearance, accordingly the level of LRP1 expression is critical for the AD progression (Asadi et al., 2021[9]; Van Gool et al., 2019[119]). Studies showed that LRP1 levels are increased in the AD brain (Matsui et al., 2007[91]). On the other hand, LRP1-AS is the natural antisense transcript of LRP1 gene (Bruno et al., 2010[18]).Yamanaka et al. observed that overexpression of LRP1-AS attenuated the LRP1 expression. It was clearly indicated that LRP1-AS negatively regulated the LRP1 gene expression at both the mRNA and protein levels (Yamanaka et al., 2015[129]). It directly binds to the high mobility group box 2 (Hmgb2) and prevents the activity of Hmgb2 to enhance the LRP1 transcription (Matsui et al., 2007[91]).

## lncRNAs in Synaptic and Neuron Plasticity

### BC200

One of the neuropathological features of AD is synaptic dysfunction that occurs in the first stages of the disorder (Kim and Song, 2018[66]; Strobel et al., 2015[115]). It has been demonstrated that the failure of synaptic plasticity is the foundation of memory deficits in AD patients (Shankar et al., 2008[110]; Singh et al., 2016[112]). Synaptic dysfunction occurs in the various cortical circuits of the brain, from the entorhinal cortex to the hippocampus (Amid et al., 2001[5]). Implication of the lncRNA BC1/BC200 in the synaptic plasticity has been reported in previous studies (Labzin et al., 2018[70]; Lin et al., 2008[77]; Wang et al., 2002[122]). BC200 lncRNA is a small untranslated RNA that selectively targets eIF4A activity in the somatodendritic domains of neurons, modulates protein synthesis in the postsynaptic dendritic microdomains and contributes to the maintenance of long-term synaptic plasticity (Li et al., 2018[74]; Wang et al., 2002[122]; Wu et al., 2013[128]). In one postmortem study, Mus et al., reported that expression of the dendritic BC200 RNA was differentially regulated in the normal aging and AD. They found that during normal aging, BC200 RNA level was reduced in the synaptic regions of the hippocampus, while, compared with the age-matched normal brains, BC200 RNA was significantly upregulated in the AD, and its level was significantly increased with the severity of the disease (Mus et al., 2007[96]). Recently, Li et al. showed that the treatment of an AD cell model with Aβ1-42 increased BC200 and BACE1 expression, and knockdown of BC200 rescued this Aβ1-42-mediated dysfunction. They also showed that BC200 might be a potent positive regulator of BACE1, and could reduce apoptosis and increase viability of the AD cells (Li et al., 2018[74]). Remarkably, BC200 plasma levels have recently been reported to be higher in AD patients, implying its potential as a blood-based biomarker for early diagnosis of AD (Khodayi et al., 2022[64]).

### BDNF-AS

Brain-derived neurotrophic factor (BDNF), is widely synthesized in the central nervous system, has a physiological role in synaptic plasticity and survival of neurons (Gu et al., 2018[46]; Zagrebelsky et al., 2018[135]). In addition, BDNF can prevent neuronal function loss, facilitates the regeneration and differentiation of injured neurons (Huang et al., 2017[55]; Li et al., 2017[73]). Previous studies indicated that the reduced mRNA and protein levels of BDNF in the serum and human brain are closely associated with the pathogenesis of AD (Azizi Aghali et al., 2018[11]; Buchman et al., 2016[19]; Holsinger et al., 2000[52]). Consistently, *in vitro *and* in vivo* experiments revealed that BDNF has potential protective effects against the neurotoxicity and learning deficits induced by Aβ peptides (Jiao et al., 2016[61]; Zhang et al., 2015[140]). BDNF gene delivery in Aβ precursor protein (APP)-transgenic mice showed a compensatory event to slow down the cognitive decline (Nagahara et al., 2013[97], 2009[98]). BDNF-AS is an antisense lncRNA of BDNF, located on the opposite strand of the BDNF on chromosome 11, and can negatively regulate the BDNF level both *in vivo* and *in vitro* (Modarresi et al., 2012[93]). Guo et al. reported that silencing of BDNF-AS diminished the Aβ25-35-induced neurotoxicity of PC12 cells and suppressed the cell apoptosis and oxidative stress (Guo et al., 2018[47]). It was reported that BDNF-AS level was significantly elevated in the peripheral blood of AD patients (Azizi Aghali et al., 2018[11]; Ding et al., 2022[29]), and that elevated expression levels of BDNF-AS might be related to the cognitive decline of AD patients (Ding et al., 2022[29]). More recently, Ding et al. reported that BDNF-AS could promote neurotoxicity in an *in vitro* model of AD by promoting the expression of BACE1, thereby resulting in the amyloid deposition. 

### GDNFOS

Glial cell line-derived neurotrophic factor (GDNF) is an important biologically active trophic factor for development and survival of the midbrain dopaminergic neurons (Airavaara et al., 2011[3]; Lin et al., 1993[78]). However, its role is not specific for dopaminergic neurons. GDNF is also involved in neurite branching, synaptic plasticity and increase of neural stem cell migration and differentiation (Airaksinen and Saarma, 2002[2]). A recent report indicated that GDNF levels are significantly increased in cerebrospinal fluid and decreased in serum in patients with early AD (Straten et al., 2009[114]). A cis-natural antisense transcript, termed GDNF opposite strand (GDNFOS), is transcribed from the opposite strand of the GDNF gene (Straten et al., 2009[114]). The GDNFOS or GDNF-AS gene has four exons that are spliced into different isoforms. GDNFOS1 and GDNFOS2 are long non-coding RNAs, with no obvious ORF. GDNFOS1 can pair to the 5ʹ-UTR of the GDNF, but GDNFOS2 has no overlapping with the GDNF transcript. GDNFOS3 transcript is not lncRNA, contains an ORF and has potential for protein translation with no known homologs in GenBank. Dysregulation of human GDNF and GDNFOS may contribute to vulnerability to neurodegenerative diseases (Airavaara et al., 2011[3]). However, more evidence is required to elucidate the relevance between GDNF-AS and GDNF mRNA as well as the relationship with AD pathogenesis. 

## lncRNAs in Neuroinflammation

### 17A 

LncRNA 17A is a 159-nt lncRNA which is transcribed by RNA polymerase III and maps to an intronic region of the GABA receptor gene (Zhou et al., 2014[147]). It has been shown that overexpression of 17A leads to the production of alternative splicing GABA receptor B (GABAB) and formation of a nonfunctional heterodimeric receptor (Gavazzo et al., 2013[41]). GAGA(B2) receptors significantly affect the GABA-B signaling pathway by inhibition of intracellular 3′-5′-cyclic adenosine monophosphate accumulation and the activation of specific potassium channels (Massone et al., 2011[90]). The inflammatory stimulations in the brain of AD patients can activate the expression of 17A and increase the secretion of Aβ and the Aβ42/Aβ40 ratio (Massone et al., 2011[90]). Wang and collaborators showed that LncRNA 17A expression was involved in cellular functions and biological processes of neuroblastoma cells in an AD cell model, indicating that it could directly or indirectly be implicated in the progression of AD (Wang et al., 2019[125]).

### NDM 29

Neuroblastoma differentiation marker 29 (NDM29), a RNA pol II-transcribed lncRNA, mapped to a genomic region of chromosome 11, and its synthesis is regulated by an extragenic type 3 promoter (Massone et al., 2012[89]). NDM29 was actively transcribed in cells of the central nervous system (Gavazzo et al., 2011[42]). Massone and collaborators reported that high levels of NDM29 transcripts induced APP synthesis, and subsequently the level of Aβ and the ratio of Aβ-42/Aβ-40 were increased. They also found that the expression of NDM29 was induced by inflammatory stimuli and inhibited by anti-inflammatory drugs (Massone et al., 2012[89]). Furthermore, this lncRNA was abnormally expressed in the cerebral cortex of AD patients, demonstrating that NDM29 is linked to AD pathology (Massone et al., 2012[89]). 

## lncRNA in Neuronal Differentiation

### Sox2OT 

Sox2 is a component of the core transcriptional regulatory network that is essential for maintaining pluripotency of stem cells and neurogenesis (Sarlak et al., 2016[108]). Sox2 overlapping transcript (Sox2OT) as an evolutionarily conserved transcript among vertebrates, has been linked to cognitive impairment in a mouse model of Alzheimer's disease (Amaral et al., 2009[4]; Fantes et al., 2003[36]). Knauss et al. recently found that Sox2OT is expressed in developing mouse cerebral cortex, where it promotes neuronal differentiation and neurogenesis by repressing Sox2 in neuronal progenitors (Knauss et al., 2018[67]). Furthermore, whole genome microarray analysis indicated that Sox2OT is differentially expressed in early and late stages of the disease in an AD model mouse, suggesting that Sox2OT might be related to the AD pathobiology (Arisi et al., 2011[8]).

## lncRNAs in Apoptosis of Neurons

### EBF3-AS 

Recently, Magistri et al. used RNA sequencing in order to discover significant alterations in expression profile of the lncRNAs from AD brains and found that lncRNA EBF3-AS is abundantly expressed in the brain of late-onset AD cases compared to control individuals (Magistri et al., 2015[85]). Furthermore, a recent study reported that the expression of early B cell factor 3 (EBF3) and lncRNA EBF3-AS were up-regulated in hippocampus of APP/PS1 transgenic mice (Gu et al., 2018[46]). They reported that EBF3-AS knockdown by siRNA could inhibit the apoptosis induced by Aβ25-35 and okadaic acid (OA) in mouse model of AD. Aβ25-35 and okadaic acid increased the expression of EBF3. They observed that lncRNA EBF3-AS stimulated the EBF3 expression and promoted neuron apoptosis in AD brain (Gu et al., 2018[46]). These evidences suggested that EBF3-AS might be considered as a new therapeutic target for the treatment of AD. 

## Other Related lncRNAs

Lee and colleagues identified 47 upregulated and 158 downregulated lncRNAs in rat model of AD compared with healthy controls using microarray analysis (Lee et al., 2015[72]). Moreover, it was reported that lncRNAs AD-linc1, AD-linc2 and HAO2-AS are upregulated in the hippocampus of the patients with AD, which indicated that these lncRNAs might have key functions in AD (Magistri et al., 2015[85]). Another notable lncRNA associated with AD is n336934, which is involved in the cholesterol homeostasis pathway. Disruption of cholesterol metabolism is a feature of AD pathogenesis (Hannaoui et al., 2014[48]). Moreover, lncRNA NAT-Rad18 is upregulated in rat cortical neurons after Aβ treatment. Therefore, the highly upregulated lncRNA NAT-Rad18 may reduce the ability of neurons to respond to DNA damage stressors in AD pathogenesis (Parenti et al., 2007[102]).

## MicroRNAs in AD

MicroRNAs (miRNAs) are endogenous ~22 nucleotide non-coding RNAs, responsible for direct posttranscriptional regulation of gene expression, either by targeting mRNA degradation or by inhibition of protein translation at both mRNA and protein levels (Kou et al., 2020[68]). After transcription, the primary microRNA transcripts are cleaved to precursor (pre) microRNAs by Drosha (Denli et al., 2004[27]), and following their export to the cytoplasm, the pre-microRNAs are processed by Dicer to generate mature microRNAs (Okada et al., 2009[101]). The microRNAs that now are incorporated into a ribonucleoprotein complex named RNA-induced silencing complex (RISC), guides the complex to the target mRNAs (O'Brien et al., 2018[100]; Zhang et al., 2004[137]). Recent studies have indicated that miRNAs are involved in the most critical biological processes, including proliferation, differentiation, development, and apoptosis (Bartel, 2009[15]; Esquela-Kerscher and Slack, 2006[33]). Specifically, miRNAs are widely found within the nervous system where they play key roles during neuronal differentiation, synaptogenesis, and plasticity (Goodall et al., 2013[45]). This has been the clue for considering miRNAs as crucial molecules to be studied in AD, and nowadays, dysfunction of miRNAs in AD is increasingly recognized (Ramakrishna and Muddashetty, 2019[105]). miRNAs that contribute to the Aβ plaque production and tau phosphorylation as the main pathologic hallmarks of AD, could play crucial roles in the AD pathobiology. Specifically, some miRNAs that have been known to be implicated in the regulation of BACE1, APP and tau expression and processing, and in the tau phosphorylation, have been outlined in Table 2(Tab. 2) (References in Table 2: Barbato et al., 2020[13]; Barros-Viegas et al., 2020[14]; Chopra et al., 2021[23]; Du et al., 2021[31]; Gong et al., 2017[44]; Hébert et al., 2008[49]; Hernandez-Rapp et al., 2016[51]; Kou et al., 2020[68]; Liu et al., 2012[80]; Long et al., 2014[82]; Ma et al., 2017[84]; Wang et al., 2008[124]; Yang et al., 2015[131]; Zhang et al., 2014[138], 2016[142]; Zhu et al., 2012[150]) and will be discussed in more details through the following sections.

### miRNAs involved in regulation of the BACE1 expression 

β-site amyloid precursor protein-cleaving enzyme1 (BACE1), a rate-limiting enzyme involved in the formation of Aβ, plays a crucial role in AD progression (Maoz et al., 2017[87]; Zong et al., 2011[152]). Several miRNAs that are potentially involved in the regulation of BACE1 expression appeared to be downregulated in AD. For instance, miR-9, miR-29c, miR-29a/b-1, miR-101, miR-107, miR-298, and miR-15b exhibit reduced expression in patients or model animals with AD and contribute to the elevated Aβ formation (Gong et al., 2017[44]; Hébert et al., 2008[49]; Wang et al., 2008[124]; Yang et al., 2015[131]; Zhang et al., 2021[143]). On the other hand, miR-339-5p was found to be significantly downregulated in the brain of AD patients compared with the age-matched healthy controls. Further analysis indicated that miR-339-5p negatively regulates BACE1 level by targeting the 3^′^-UTR region of the BACE1 to reduce Aβ accumulation, which makes it as a novel drug target (Long et al., 2014[82]). Similarly, both miR-195 and miR-135b negatively regulate expression level of BACE1 by binding to the 3′-UTR of the BACE1 mRNA (Zhang et al., 2016[142]; Zhu et al., 2012[150]). Other miRNA that was found to be involved in the Aβ regulation is miR-188-3p. It was negatively correlated with the BACE1 abundance and Aβ production, and improved the long-term synaptic plasticity in an APP transgenic mice (Zhang et al., 2014[138]). Furthermore, Hébert et al. reported that expression of the miR-29a/b-1 was significantly low in AD brain but the BACE1 protein level was significantly high, and the decreased level of the miR-29a/b-1 in human cell culture could promote the Aβ production (Hébert et al., 2008[49]). In another study, Yang et al. found that miR-29c, a member of the miR-29 family, plays a role in the regulation of BACE1 abundance by targeting its 3′-UTR both in human and mouse cell lines. Interestingly, upregulation of miR-29c in the hippocampus of SAMP8 mice improved spatial memory (Yang et al., 2015[131]). Du et al. observed a decreased expression of the miRNA cluster miR-149 in individuals with AD as well as in an AD cell model. This study identified BACE1 as a direct target of miR-149, and showed that the miR-149 deficiency in mice could led to the increased Aβ accumulation. Furthermore, the overexpression of miR-149 in Aβ-treated SH-SY5Y cells inhibited the Aβ accumulation and promoted the neuronal viability (Du et al., 2021[31]). In summary, several miRNAs play important roles in regulating BACE1 protein levels and comprehensive understandings of the complex network of regulatory microRNAs that suppress the BACE1 production may lead to the discovery of breakthrough treatment strategies for AD.

### miRNAs in regulation of APP expression

Mounting evidence has shown that the regulation of APP expression by miRNAs could affect production and accumulation of Aβ, resulting in neurotoxicity and synaptic failure (Improta-Caria et al., 2020[58]; Patel et al., 2008[103]). Different miRNAs have been reported to be involved in the regulation of APP gene expression and thus the accumulation of fibrillar Aβ. For example, hsa-mir-106a and hsa-mir-520c were negatively correlated with the endogenous APP levels in HEK-293 cells and showed regulatory effects by binding to the 3′-UTR of APP (Kou et al., 2020[68]; Patel et al., 2008[103]). Moreover, miR-16 was also found to target APP mRNA and suppress APP production, and cause accumulation of APP protein in SAMP8 model mice (Liu et al., 2012[80]). Additionally, another study demonstrated that inhibition of miR-16 in primary hippocampal neurons decreased the cell viability in a cellular AD model (Zhang et al., 2015[136]). Similarly, the binding of miR-101, miR-31, and miR-17 to the 3^′^-UTR of the APP mRNA resulted in decreased level of APP in patients with AD (Barros-Viegas et al., 2020[14]; Kou et al., 2020[68]). Interestingly, the high level of miR-31 reduced the levels of APP and BACE1 mRNAs in the hippocampus of 17-months-old AD triple-transgenic (3xTg-AD) female mice, and improved memory deficits and cognitive inflexibility (Barros-Viegas et al., 2020[14]).

### miRNAs in tau expression and tau phosphorylation

Abnormal accumulation of intraneuronal filaments formed by tau proteins is the most common pathological feature in AD (Muralidar et al., 2020[95]). Several miRNAs have been reported to be related to tauopathy. For instance, the miR-132/212 cluster is frequently downregulated in Ad and has been shown to regulate tau *in vitro *and* in vivo* (Hernandez-Rapp et al., 2016[51]). Smith et al. reported that miR-132/212 deficiency in mice leads to increased tau expression, phosphorylation and aggregation (Smith et al., 2015[113]). It has been reported that miRNAs can affect tau phosphorylation and subsequently modulate tangle formation. miR-15 may induce tau hyperphosphorylation through ERK1 in Neuro2a cells (Hébert et al., 2010[50]). Studies have demonstrated that miR-106b is downregulated in sporadic AD patients as well as in the human neuroblastoma SH-SY5Y cells, and it can inhibit Aβ42-induced tau phosphorylation at the site of Tyr18 (Liu et al., 2016[81]). Moreover, one study has found that overexpression of miR-125b could lead to cell apoptosis and tau hyperphosphorylation by activating the cyclin-dependent kinase 5 (Banzhaf‐Strathmann et al., 2014[12]).

## Conclusions and Perspectives

In this review, we summarized the relationship between ncRNAs and AD pathogenesis based on recent evidence. Multiple studies have shown the critical role of ncRNAs in various biological processes, including cell growth, apoptosis, and differentiation, indicating that they might be involved in the occurrence and progress of neurodegenerative diseases including AD (Barbato et al., 2020[13]; Zhou et al., 2019[148]). Recent progress in biotechnologies such as high-throughput sequencing, mouse modeling, cell lines treated with different Aβ peptides, and human biological samples such as brain, CSF, and serum have provided important insights into the biological function and clinical relevance of ncRNAs in AD (Bruno et al., 2020[17]; Zhao et al., 2019[146]). These ncRNAs were associated with diverse processes in the brain underlying the pathogenesis of AD, including formation of Aβ plaques, the phosphorylation of Tau, synaptic dysfunction, and neuroinflammation (Zhang et al., 2021[143]). It is necessary and urgent to seek reliable and effective biomarkers for early diagnosis of AD, and timely detection of the disease can offer the opportunities for early intervention to potentially delay or prevent the pathological process of AD. In recent years, most studies suggest that ncRNAs are desirable candidates as diagnostic markers of preclinical or clinical AD due to their easy accessibility and stability throughout storage and handling. Understanding the regulatory function and molecular mechanisms of ncRNAs in AD pathophysiology will allow the development of new therapies and diagnostic markers for AD. In conclusion, although the role and impact of ncRNAs in the pathogenesis of AD remain partially unknown, but promise to open more avenues to early diagnosis and treatment of AD.

## Declaration

### Highlights

- Non-coding RNAs play crucial roles in the pathogenesis of Alzheimer's disease.
- Non-coding RNAs (ncRNAs) can be used as potential therapeutic targets and biomarkers.
- Reliable biomarkers for early diagnosis are required to halt the disease development and improve the effects of therapeutic strategies.

### Acknowledgments

Authors appreciate the Cognitive Sciences and Technologies Council of Iran for supporting this work.

### Funding

This work was supported by the Cognitive Sciences and Technologies Council of Iran, grant number 6300.

### Competing interests

The authors have no relevant financial or non-financial interests to disclose.

### Author contributions

Majid Khodayi-Shahrak performed research and wrote the manuscript; Mohammad Khalaj-Kondori supervised research, reviewed and edited the manuscript; Mohammad Ali Hosseinpour Feizi reviewed and edited the manuscript; Mahnaz Talebi reviewed and edited the manuscript. 

## Figures and Tables

**Table 1:**
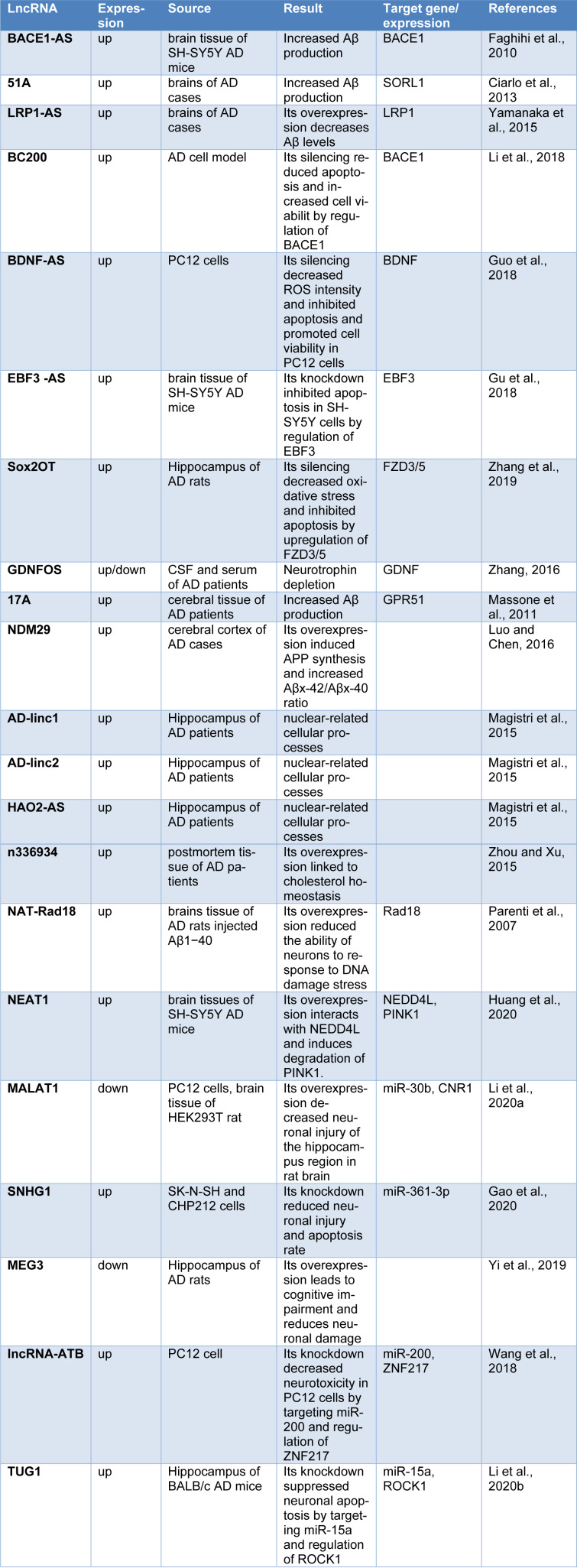
The lncRNAs involved in Alzheimer's disease

**Table 2:**
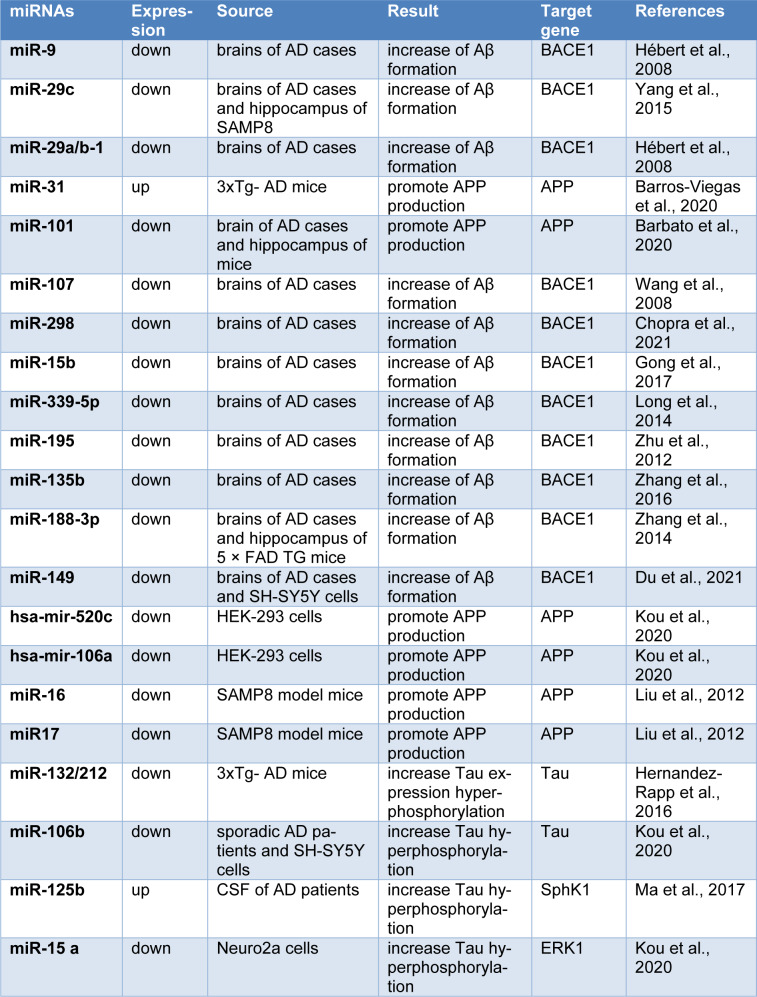
A list of miRNAs that are involved in the regulation of BACE1, APP and tau processing
